# Bis{2-[(*E*)-benzyl­imino­meth­yl]-4-methyl­phenolato-κ^2^ *N*,*O*}nickel(II)

**DOI:** 10.1107/S1600536809026658

**Published:** 2009-07-15

**Authors:** Su-Zhen Chen, Dong-Guo Xia

**Affiliations:** a State Key Laboratory Base of Novel Functional Materials and Preparation Science, Institute of Solid Materials Chemistry, Faculty of Materials Science and Chemical Engineering, Ningbo University, Ningbo 315211, People’s Republic of China

## Abstract

In the title complex, [Ni(C_15_H_14_NO)_2_], the Ni^II^ atom is located on an inversion centre and is coordinated by two O and two N atoms from two symmetry-related bidentate Schiff base ligands in a slightly distorted square-planar geometry. The phenyl and benzene rings in the ligand mol­ecule form a dihedral angle of 72.79 (8)°.

## Related literature

For the synthesis of 2-[(*E*)-(benzyl­imino)meth­yl]-4-methyl­phenol, see: Cohen *et al.* (1964 ▶). For the structure of a related Zn complex, see: Rodriguez de Barbarin *et al.* (1994 ▶).
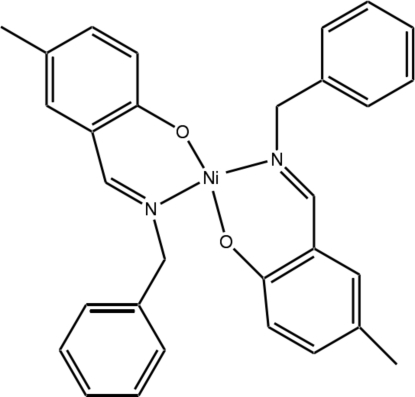


## Experimental

### 

#### Crystal data

[Ni(C_15_H_14_NO)_2_]
*M* *_r_* = 507.26
Monoclinic, 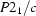

*a* = 13.7182 (15) Å
*b* = 10.5842 (11) Å
*c* = 8.6716 (9) Å
β = 107.593 (1)°
*V* = 1200.2 (2) Å^3^
*Z* = 2
Mo *K*α radiation
μ = 0.84 mm^−1^
*T* = 296 K
0.37 × 0.29 × 0.24 mm

#### Data collection

Bruker SMART APEXII diffractometer
Absorption correction: multi-scan (**SADABS**; Sheldrick, 2000 ▶) *T*_min_ = 0.751, *T*_max_ = 0.819
10296 measured reflections
2765 independent reflections
2303 reflections with *I* > 2σ(*I*)
*R*_int_ = 0.029

#### Refinement

*R*[*F* ^2^ > 2σ(*F* ^2^)] = 0.030
*wR*(*F* ^2^) = 0.081
*S* = 1.04
2765 reflections
161 parameters
H-atom parameters constrained
Δρ_max_ = 0.35 e Å^−3^
Δρ_min_ = −0.24 e Å^−3^

### 

Data collection: *APEX2* (Bruker, 2007 ▶); cell refinement: *SAINT* (Bruker, 2007 ▶); data reduction: *SAINT*; program(s) used to solve structure: *SHELXS97* (Sheldrick, 2008 ▶); program(s) used to refine structure: *SHELXL97* (Sheldrick, 2008 ▶); molecular graphics: *SHELXTL* (Sheldrick, 2008 ▶); software used to prepare material for publication: *SHELXTL*.

## Supplementary Material

Crystal structure: contains datablocks global, I. DOI: 10.1107/S1600536809026658/is2434sup1.cif

Structure factors: contains datablocks I. DOI: 10.1107/S1600536809026658/is2434Isup2.hkl

Additional supplementary materials:  crystallographic information; 3D view; checkCIF report

**Fig. 1.**
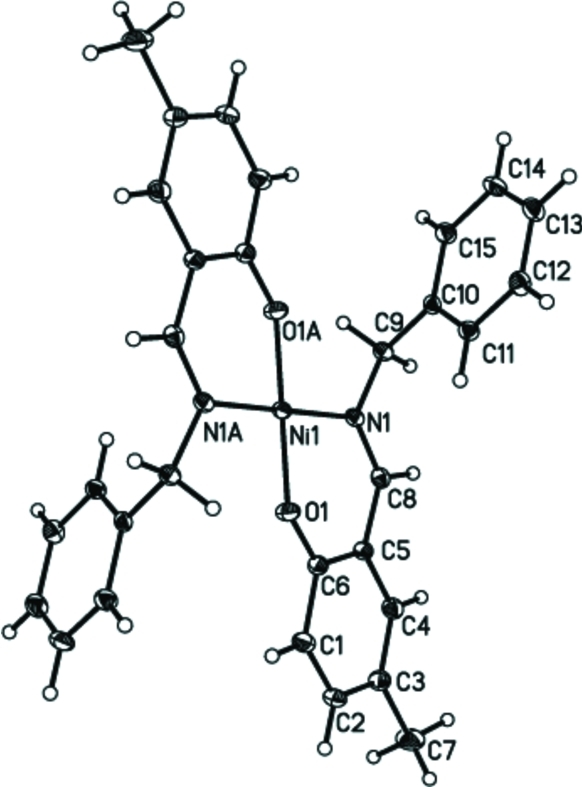
The structure of (I), showing displacement ellipsoids drawn at the 30% probability level [symmetry code: (A) -x + 2, -y + 1, -z].

Enhanced figure: interactive version of Fig. 1

## Figures and Tables

**Table:** 

Ni1—O1	1.8294 (12)
Ni1—N1	1.9242 (14)

**Table:** 

O1^i^—Ni1—N1	87.01 (6)
O1—Ni1—N1	92.99 (6)

Symmetry code: (i) 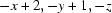.

## supplementary crystallographic information

### Comment

Schiff bases have played an important role in the development of coordination chemistry as they readily form stable complexes with most of the transition metals (Rodriguez de Barbarin *et al.*, 1994). Salicylaldehyde and its derivatives are useful carbonyl precursors for the synthesis of a large variety of Schiff bases. Here we report on a new Ni(II) complex, (I).

The molecular structure of (I) as illustrated in Fig. 1 has the Ni^2+^ center in a square geometry as it its coordinated by two O atoms and two N atoms from two 2-[(*E*)-(benzylimino)methyl]-4-methylphenol bidentate chelating ligands. The bond lengths and bond angles in (I) are within normal ranges. The Ni1—O1 distance of 1.8294 (12) Å is shorter than the distance of Ni1—N1 [1.9242 (14) Å] (Table 1). The dihedral angle between the plane of O1/N1/Ni1 and the adjacent phenol ring is 10.91 (9)°.

### Experimental

1 mmol of NiCl_2_.6H_2_O (0.240 g) were added to a 15 ml ethanol solution containing 2 mmol (0.450 g) 2-[(*E*)-(benzylimino)methyl]-4-methylphenol. The resulting mixture was stirred for about 0.5 h. The slow vaporization of the solvent yielded after about 6 d dark green single crystals. Yield: 70.2%. Calcd. for C_30_H_28_NiN_2_O_2_: C 71.03, H 5.56, N 5.52; Found: C 71.34, H 5.60, N 5.46%.

Synthesis of the ligand 2-[(*E*)-(benzylimino)methyl]-4-methylphenol: Phenylmethanamine and 5-methylsalicylaldehyde (1:1) were dissolved in ethanol and the solution was refluxed for 3 h. After evaporation, a crude product was recrystallized twice from ethanol to give a pure yellow product (Cohen *et al.*, 1964).

### Refinement

All H atoms were located in a difference Fourier map. H atoms of the C—H groups were then  placed in geometrically idealized positions and constrained to ride on their parent atoms with C—H = 0.93, 0.96 or 0.97 Å, and with *U*_iso_(H) = 1.2*U*_eq_(C).

### Crystal data

**Table:**

[Ni(C_15_H_14_NO)_2_]	*F*(000) = 532.0
*M**_r_* = 507.26	*D*_x_ = 1.404 Mg m^−^^3^
Monoclinic, *P*2_1_/*c*	Mo *K*α radiation, λ = 0.71073 Å
Hall symbol: -P 2ybc	Cell parameters from 3741 reflections
*a* = 13.7182 (15) Å	θ = 3.1–27.5°
*b* = 10.5842 (11) Å	µ = 0.84 mm^−^^1^
*c* = 8.6716 (9) Å	*T* = 296 K
β = 107.593 (1)°	Block, dark green
*V* = 1200.2 (2) Å^3^	0.37 × 0.28 × 0.24 mm
*Z* = 2	

### Data collection

**Table:**

Bruker SMART APEXII diffractometer	2765 independent reflections
Radiation source: fine-focus sealed tube	2303 reflections with *I* > 2σ(*I*)
graphite	*R*_int_ = 0.029
φ and ω scans	θ_max_ = 27.5°, θ_min_ = 2.5°
Absorption correction: multi-scan (*SADABS*; Sheldrick, 2000)	*h* = −16→17
*T*_min_ = 0.751, *T*_max_ = 0.819	*k* = −13→12
10296 measured reflections	*l* = −11→11

### Refinement

**Table:**

Refinement on *F*^2^	Primary atom site location: structure-invariant direct methods
Least-squares matrix: full	Secondary atom site location: difference Fourier map
*R*[*F*^2^ > 2σ(*F*^2^)] = 0.030	Hydrogen site location: inferred from neighbouring sites
*wR*(*F*^2^) = 0.081	H-atom parameters constrained
*S* = 1.04	*w* = 1/[σ^2^(*F*_o_^2^) + (0.0383*P*)^2^ + 0.6191*P*] where *P* = (*F*_o_^2^ + 2*F*_c_^2^)/3
2765 reflections	(Δ/σ)_max_ = 0.001
161 parameters	Δρ_max_ = 0.35 e Å^−^^3^
0 restraints	Δρ_min_ = −0.24 e Å^−^^3^

### Special details

**Table:**

Geometry. All e.s.d.'s (except the e.s.d. in the dihedral angle between two l.s. planes) are estimated using the full covariance matrix. The cell e.s.d.'s are taken into account individually in the estimation of e.s.d.'s in distances, angles and torsion angles; correlations between e.s.d.'s in cell parameters are only used when they are defined by crystal symmetry. An approximate (isotropic) treatment of cell e.s.d.'s is used for estimating e.s.d.'s involving l.s. planes.
Refinement. Refinement of *F*^2^ against ALL reflections. The weighted *R*-factor *wR* and goodness of fit *S* are based on *F*^2^, conventional *R*-factors *R* are based on *F*, with *F* set to zero for negative *F*^2^. The threshold expression of *F*^2^ > σ(*F*^2^) is used only for calculating *R*-factors(gt) *etc*. and is not relevant to the choice of reflections for refinement. *R*-factors based on *F*^2^ are statistically about twice as large as those based on *F*, and *R*- factors based on ALL data will be even larger.

### Fractional atomic coordinates and isotropic or equivalent isotropic displacement parameters (Å^2^)

**Table:**

	*x*	*y*	*z*	*U*_iso_*/*U*_eq_	
Ni1	1.0000	0.5000	0.0000	0.01763 (10)	
O1	1.13609 (9)	0.46366 (12)	0.08566 (15)	0.0256 (3)	
N1	1.01028 (10)	0.65072 (13)	0.12801 (16)	0.0188 (3)	
C1	1.31316 (14)	0.49471 (18)	0.1963 (2)	0.0264 (4)	
H1A	1.3255	0.4178	0.1538	0.032*	
C2	1.39446 (14)	0.56674 (19)	0.2854 (2)	0.0283 (4)	
H2A	1.4606	0.5370	0.3017	0.034*	
C3	1.38044 (13)	0.68377 (19)	0.3525 (2)	0.0282 (4)	
C4	1.28159 (13)	0.72439 (18)	0.3268 (2)	0.0257 (4)	
H4A	1.2704	0.8009	0.3714	0.031*	
C5	1.19653 (12)	0.65377 (16)	0.23490 (19)	0.0205 (3)	
C6	1.21133 (13)	0.53616 (17)	0.16842 (19)	0.0215 (3)	
C7	1.47135 (16)	0.7606 (2)	0.4500 (3)	0.0432 (5)	
H7A	1.4488	0.8236	0.5112	0.065*	
H7B	1.5029	0.8009	0.3781	0.065*	
H7C	1.5199	0.7058	0.5225	0.065*	
C8	1.09560 (13)	0.70026 (16)	0.21563 (19)	0.0207 (3)	
H8A	1.0906	0.7736	0.2718	0.025*	
C9	0.91662 (12)	0.71815 (15)	0.13304 (19)	0.0200 (3)	
H9A	0.8749	0.7359	0.0232	0.024*	
H9B	0.9361	0.7984	0.1878	0.024*	
C10	0.85332 (12)	0.64504 (15)	0.21800 (18)	0.0184 (3)	
C11	0.89270 (13)	0.54673 (16)	0.3250 (2)	0.0211 (3)	
H11A	0.9600	0.5209	0.3427	0.025*	
C12	0.83210 (14)	0.48676 (17)	0.4056 (2)	0.0257 (4)	
H12A	0.8592	0.4209	0.4766	0.031*	
C13	0.73212 (15)	0.52398 (18)	0.3814 (2)	0.0288 (4)	
H13A	0.6919	0.4838	0.4359	0.035*	
C14	0.69245 (14)	0.6218 (2)	0.2752 (2)	0.0314 (4)	
H14A	0.6252	0.6477	0.2584	0.038*	
C15	0.75229 (13)	0.68172 (18)	0.1936 (2)	0.0258 (4)	
H15A	0.7246	0.7470	0.1219	0.031*	

### Atomic displacement parameters (Å^2^)

**Table:**

	*U*^11^	*U*^22^	*U*^33^	*U*^12^	*U*^13^	*U*^23^
Ni1	0.01551 (16)	0.01832 (17)	0.01976 (15)	−0.00024 (12)	0.00637 (11)	−0.00090 (11)
O1	0.0169 (6)	0.0255 (7)	0.0320 (7)	0.0001 (5)	0.0037 (5)	−0.0069 (5)
N1	0.0184 (7)	0.0189 (7)	0.0210 (6)	0.0005 (5)	0.0087 (5)	0.0025 (5)
C1	0.0206 (9)	0.0296 (10)	0.0299 (9)	0.0024 (7)	0.0089 (7)	−0.0021 (7)
C2	0.0169 (8)	0.0387 (11)	0.0307 (9)	−0.0013 (8)	0.0092 (7)	0.0004 (8)
C3	0.0205 (9)	0.0366 (11)	0.0276 (9)	−0.0085 (8)	0.0073 (7)	−0.0024 (8)
C4	0.0247 (9)	0.0262 (10)	0.0278 (8)	−0.0048 (7)	0.0103 (7)	−0.0020 (7)
C5	0.0182 (8)	0.0229 (9)	0.0211 (7)	−0.0031 (6)	0.0072 (6)	0.0012 (6)
C6	0.0194 (8)	0.0247 (9)	0.0208 (8)	−0.0026 (6)	0.0066 (6)	0.0005 (6)
C7	0.0231 (10)	0.0525 (14)	0.0527 (12)	−0.0122 (10)	0.0094 (9)	−0.0174 (11)
C8	0.0239 (9)	0.0184 (8)	0.0222 (8)	−0.0025 (6)	0.0103 (7)	0.0003 (6)
C9	0.0195 (8)	0.0176 (8)	0.0240 (8)	0.0026 (6)	0.0082 (6)	0.0013 (6)
C10	0.0197 (8)	0.0184 (8)	0.0181 (7)	0.0005 (6)	0.0074 (6)	−0.0023 (6)
C11	0.0194 (8)	0.0215 (8)	0.0231 (8)	0.0034 (7)	0.0073 (6)	0.0012 (6)
C12	0.0301 (10)	0.0238 (9)	0.0247 (8)	0.0023 (7)	0.0105 (7)	0.0043 (7)
C13	0.0286 (10)	0.0333 (11)	0.0289 (9)	−0.0050 (8)	0.0153 (8)	0.0008 (7)
C14	0.0191 (9)	0.0446 (12)	0.0334 (9)	0.0053 (8)	0.0123 (7)	0.0035 (8)
C15	0.0230 (9)	0.0303 (10)	0.0248 (8)	0.0068 (7)	0.0084 (7)	0.0055 (7)

### Geometric parameters (Å, °)

**Table:**

Ni1—O1^i^	1.8294 (12)	C7—H7A	0.9600
Ni1—O1	1.8294 (12)	C7—H7B	0.9600
Ni1—N1^i^	1.9242 (14)	C7—H7C	0.9600
Ni1—N1	1.9242 (14)	C8—H8A	0.9300
O1—C6	1.312 (2)	C9—C10	1.511 (2)
N1—C8	1.298 (2)	C9—H9A	0.9700
N1—C9	1.482 (2)	C9—H9B	0.9700
C1—C2	1.379 (3)	C10—C11	1.390 (2)
C1—C6	1.414 (2)	C10—C15	1.392 (2)
C1—H1A	0.9300	C11—C12	1.391 (2)
C2—C3	1.406 (3)	C11—H11A	0.9300
C2—H2A	0.9300	C12—C13	1.381 (3)
C3—C4	1.375 (3)	C12—H12A	0.9300
C3—C7	1.514 (3)	C13—C14	1.383 (3)
C4—C5	1.412 (2)	C13—H13A	0.9300
C4—H4A	0.9300	C14—C15	1.389 (2)
C5—C6	1.412 (2)	C14—H14A	0.9300
C5—C8	1.431 (2)	C15—H15A	0.9300
			
O1^i^—Ni1—O1	180.00 (8)	C3—C7—H7C	109.5
O1^i^—Ni1—N1^i^	92.99 (6)	H7A—C7—H7C	109.5
O1—Ni1—N1^i^	87.01 (6)	H7B—C7—H7C	109.5
O1^i^—Ni1—N1	87.01 (6)	N1—C8—C5	126.80 (16)
O1—Ni1—N1	92.99 (6)	N1—C8—H8A	116.6
N1^i^—Ni1—N1	180.00 (7)	C5—C8—H8A	116.6
C6—O1—Ni1	129.55 (12)	N1—C9—C10	113.51 (13)
C8—N1—C9	115.13 (14)	N1—C9—H9A	108.9
C8—N1—Ni1	124.64 (12)	C10—C9—H9A	108.9
C9—N1—Ni1	120.22 (10)	N1—C9—H9B	108.9
C2—C1—C6	120.95 (17)	C10—C9—H9B	108.9
C2—C1—H1A	119.5	H9A—C9—H9B	107.7
C6—C1—H1A	119.5	C11—C10—C15	118.58 (15)
C1—C2—C3	122.03 (17)	C11—C10—C9	122.97 (14)
C1—C2—H2A	119.0	C15—C10—C9	118.38 (15)
C3—C2—H2A	119.0	C10—C11—C12	120.43 (16)
C4—C3—C2	117.38 (17)	C10—C11—H11A	119.8
C4—C3—C7	121.88 (18)	C12—C11—H11A	119.8
C2—C3—C7	120.74 (17)	C13—C12—C11	120.70 (16)
C3—C4—C5	122.16 (17)	C13—C12—H12A	119.6
C3—C4—H4A	118.9	C11—C12—H12A	119.6
C5—C4—H4A	118.9	C12—C13—C14	119.18 (17)
C4—C5—C6	120.06 (16)	C12—C13—H13A	120.4
C4—C5—C8	119.30 (16)	C14—C13—H13A	120.4
C6—C5—C8	120.59 (15)	C13—C14—C15	120.46 (16)
O1—C6—C5	123.54 (16)	C13—C14—H14A	119.8
O1—C6—C1	119.03 (16)	C15—C14—H14A	119.8
C5—C6—C1	117.42 (16)	C14—C15—C10	120.65 (16)
C3—C7—H7A	109.5	C14—C15—H15A	119.7
C3—C7—H7B	109.5	C10—C15—H15A	119.7
H7A—C7—H7B	109.5		

Symmetry codes: (i) −*x*+2, −*y*+1, −*z*.
